# Red Sulphur Spring of Macon County, Tennessee—Its Medical Virtues

**Published:** 1853-10

**Authors:** Archibald M. Debow

**Affiliations:** Hartsville, Tennessee


ART. V.—*Red Sulphur Spring of Macon County, Tennessee — Its Medical Virtues.* Communicated in a Letter to Dr. YANDELL. By ARCHIBALD M. DEBOW, M.D., of Hartsville, Tenn.

DEAR SIR: About a year ago, I wrote to you, giving some account of a mineral spring in Macon county, of this State, known by the name of the Red Sulphur Spring. This spring has been much resorted to by the invalid during the present year, as also by those seeking pleasure and retirement.

The character which I felt authorized to give this spring in my communication to you has been fully sustained this season, and I have no hesitation in renewing my expression of confidence in the medicinal virtues of the water as then expressed.

I am glad, however, to have it in my power to speak of another, in the same county, possessing all the properties of the Red Sulphur Spring, and what might be considered an additional advantage, namely, that there breaks out within a few yards of it an excellent Chalybeate Spring, said by those capable of judging correctly, to be equal to the water of the Bon Air or Beersheba of the Mountain District. That this spring is strongly impregnated with iron I suppose there is no doubt, from the experiments that have been made by scientific gentlemen.

In addition, too, to the two springs — the Red Sulphur and Chalybeate — breaking out in close proximity to each other, being not more than twenty or thirty steps apart, there are two other springs in the same flat, each possessing mineral properties different somewhat from the two above named, and not more than eighty yards the extreme distance from one to either of the others. These last springs have not been analyzed, and I cannot say what may be their precise properties, though I am well satisfied they are each different the one from the other, though each and all of them may, to some extent, exhibit the same properties upon strict analysis.

I have this summer visited this watering place, known by the name of Epperson Springs, and feel no hesitation in recommending it to all such as may be afflicted with disease of the kidneys, and bladder; particularly those suffering with gravel or stone. And not only those, but persons afflicted with enlargement of the spleen, liver, or those troubled with general derangement of the digestive organs.

While the Red Sulphur water possesses active diuretic properties, and promises much in cases of dropsy, either partial or general, as cases could be given to prove, the Chalybeate is close at hand to be resorted to in due time as a tonic, and for those many forms of female disease to which tonics are so admirably adapted.

I have resorted to the same means to ascertain the solvent properties of the Epperson Spring water that I

did with the Red Sulphur spring water of which I wrote you a year ago, and find both to possess the same virtues; and here I feel authorized to say that as regards the Red Sulphur springs at both places, they are alike and capable of producing the same good effects in similar cases.

The Epperson Springs are situated in the Northwestern corner of Macon county, some ten miles from the Kentucky line, on the waters of the Big Trammel Creek, about four miles from the turnpike leading from Scottsville, Ky., to Gallatin and Nashville, in a romantic and healthy section of country, surrounded by an industrious and enterprising people. The place was orignally known as Will's Lick, a place of great resort for deer before the country became settled.

This is the first season these Springs have been opened for visitors, and such have been the benefits resulting that other and more extensive accommodations have become necessary, which will be provided during the winter and next spring, so that by the opening of the next watering season all can be made comfortable who may desire to sojourn there for a time.

My conviction is, that both the springs referred to in my former letter, and those just opened, promise great benefit to invalids, and it is in this persuasion that I have written these notices of them.

SEPTEMBER 17, 1853.

In the communication above referred to by Dr. Debow, (September No., 1852,) he expressed the belief that the water of the Red Sulphur Spring, under consideration, "causes those afflicted with gravel or stone to throw off quantities of detritus; so that though suffering intensely at the time of visiting the spring, they have in a few weeks left it either entirely cured, or very greatly relieved." He stated that of some seventeen persons laboring under calculous symptoms who resorted to the spring a year ago, there was not one who was not materially benefited by a sojourn of a few weeks. He gave the results of some rude experiments undertaken to test the solvent powers of the water when applied to urinary calculi out of the body. He says: "About the first of July last I deposited in a six-ounce vial two small stones—one of the magnesian phosphate, the other of the oxalate of lime variety. I submitted both of them to the same treatment, to see what, if any, effect would in course of time be wrought upon them. Fresh water was supplied every morning, and no friction employed except such as would necessarily arise from pouring in and out the water. In thirty-five days the magnesian phosphate was entirely dissolved; and in sixty-one days the oxalate was reduced to a fine powder. A similar result was had last year."

It is due to the author of these communications to state, that he is a physician of experience and eminence, and has had no inconsiderable practice in surgery. He has operated several times successfully for stone in the bladder. We have the utmost confidence in his candor, and would not suspect him of being easily carried away by enthusiasm. Nevertheless, while we have felt it proper to publish his account of these springs, we are constrained to say that we cannot help distrusting their lithontriptic powers, well attested as they would seem to be. We know that our friend, Dr. Debow, is fully persuded of their reality, but we all know how often practitioners have been deceived about the efficacy of new remedies. More time is required to establish the claims of these waters to such wonderful virtues than has yet expired. That patients troubled with calculous affections have been benefited by them there can be no question, and this is enough to recommend them to the valitudinary; but that they have the power to dissolve calculi in the bladder, after passing through the system, is a thing so improbable that before we can receive it we must insist upon further time and a more extended experience.

The springs, we have every reason to believe, are excellent, and, in justice to Dr. Debow, we ought to add that he has no pecuniary interest in them. We hope the proprietors will have an authoritative analysis of them made and published.—[EDS. WEST. JOUR. MED.]

